# Comparative Genomics Reveals Prophylactic and Catabolic Capabilities of *Actinobacteria* within the Fungus-Farming Termite Symbiosis

**DOI:** 10.1128/mSphere.01233-20

**Published:** 2021-03-03

**Authors:** Robert Murphy, René Benndorf, Z. Wilhelm de Beer, John Vollmers, Anne-Kristin Kaster, Christine Beemelmanns, Michael Poulsen

**Affiliations:** a University of Copenhagen, Department of Biology, Section for Ecology and Evolution, Copenhagen East, Denmark; b Leibniz Institute for Natural Product Research and Infection Biology, Hans Knöll Institute, Jena, Germany; c Department of Microbiology and Plant Pathology, Forestry and Agriculture Biotechnology Institute, University of Pretoria, Pretoria, South Africa; d Institute for Biological Interfaces (IBG 5), Karlsruhe Institute of Technology, Eggenstein-Leopoldshafen, Germany; Martin Luther University of Halle-Wittenberg Institute of Biology/Microbiology

**Keywords:** biosynthetic gene clusters, carbohydrate-active enzymes, *Macrotermitinae*, *Streptomyces*, *Actinobacteria*, *Actinomadura*, *Amycolatopsis*, *Luteimicrobium*, *Mycolicibacterium*, *Nocardia*, antimicrobial

## Abstract

*Actinobacteria*, one of the largest bacterial phyla, are ubiquitous in many of Earth’s ecosystems and often act as defensive symbionts with animal hosts. Members of the phylum have repeatedly been isolated from basidiomycete-cultivating fungus-farming termites that maintain a monoculture fungus crop on macerated dead plant substrate. The proclivity for antimicrobial and enzyme production of *Actinobacteria* make them likely contributors to plant decomposition and defense in the symbiosis. To test this, we analyzed the prophylactic (biosynthetic gene cluster [BGC]) and metabolic (carbohydrate-active enzyme [CAZy]) potential in 16 (10 existing and six new genomes) termite-associated *Actinobacteria* and compared these to the soil-dwelling close relatives. Using antiSMASH, we identified 435 BGCs, of which 329 (65 unique) were similar to known compound gene clusters, while 106 were putatively novel, suggesting ample prospects for novel compound discovery. BGCs were identified among all major compound categories, including 26 encoding the production of known antimicrobial compounds, which ranged in activity (antibacterial being most prevalent) and modes of action that might suggest broad defensive potential. Peptide pattern recognition analysis revealed 823 (43 unique) CAZymes coding for enzymes that target key plant and fungal cell wall components (predominantly chitin, cellulose, and hemicellulose), confirming a substantial degradative potential of these bacteria. Comparison of termite-associated and soil-dwelling bacteria indicated no significant difference in either BGC or CAZy potential, suggesting that the farming termite hosts may have coopted these soil-dwelling bacteria due to their metabolic potential but that they have not been subject to genome change associated with symbiosis.

**IMPORTANCE** *Actinobacteria* have repeatedly been isolated in fungus-farming termites, and our genome analyses provide insights into the potential roles they may serve in defense and for plant biomass breakdown. These insights, combined with their relatively higher abundances in fungus combs than in termite gut, suggest that they are more likely to play roles in fungus combs than in termite guts. Up to 25% of the BGCs we identify have no similarity to known clusters, indicating a large potential for novel chemistry to be discovered. Similarities in metabolic potential of soil-dwelling and termite-associated bacteria suggest that they have environmental origins, but their consistent presence with the termite system suggests their importance for the symbiosis.

## INTRODUCTION

*Actinobacteria* is a Gram-positive bacterial phylum that represents one of the largest bacterial clades. Members of the *Actinobacteria* are adapted to a range of environmental conditions and produce a variety of extracellular enzymes and natural products, many of the latter serving as drugs or drug leads (1). *Actinobacteria* associate with a diverse set of eukaryotic hosts, particularly insects, serving protective roles through the production of antimicrobials (2–7). Well-known examples include the European beewolves (genus *Philanthu*s) that host antifungal-producing species in the antennae to protect their larvae from fungal infection (8, 9), and members of the genus *Pseudonocardia* in New World fungus-farming ants that help defend from specialized mycoparasites of the ants’ fungal mutualism (10–12). *Actinobacteria* have also repeatedly been isolated from the Old World fungus-farming termite symbiosis (13–15), but their potential symbiotic roles have remained elusive.

**FIG 1:**
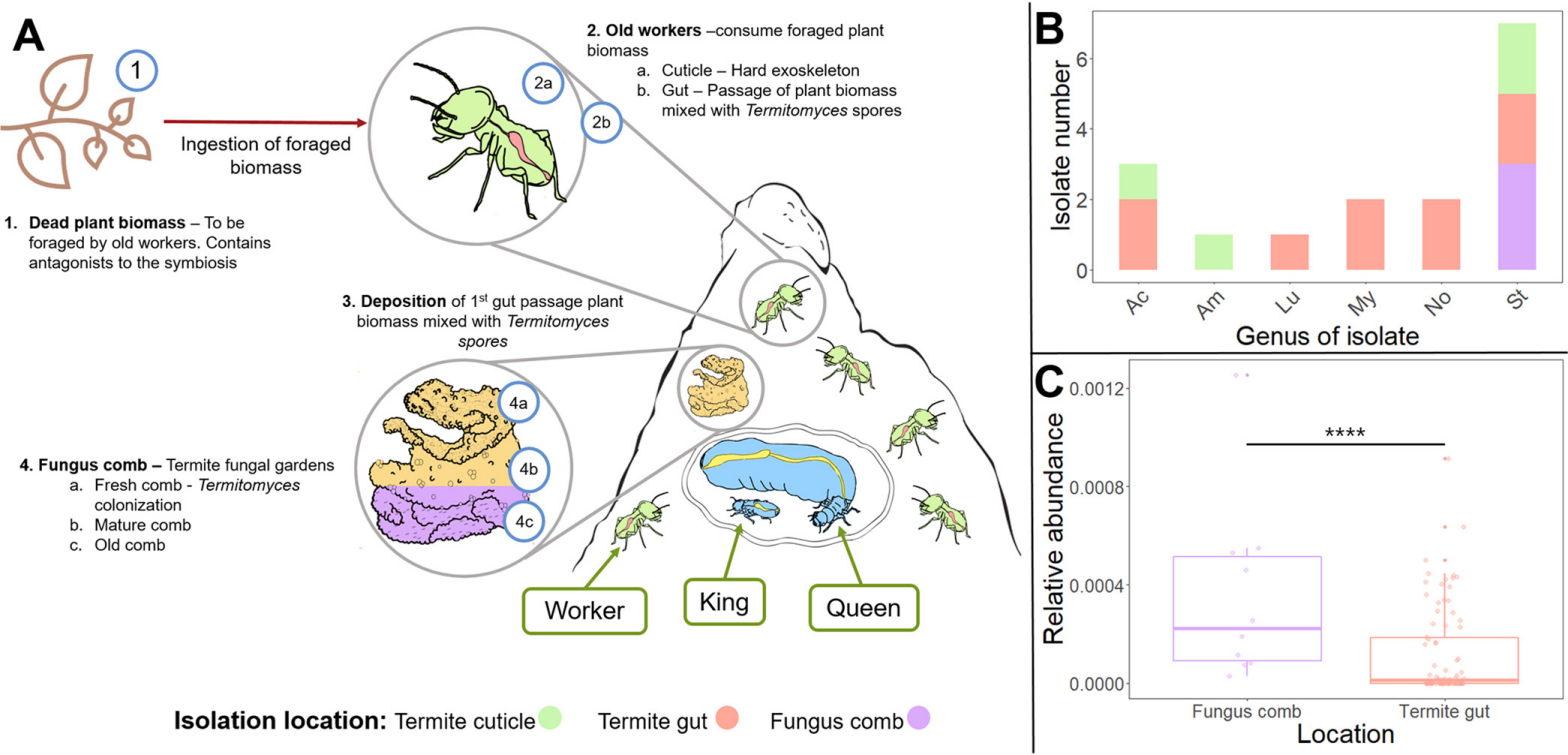
Isolation sources and sites in fungus-farming termite colonies where *Actinobacteria* could contribute to symbiosis. (A) Biomass route through the fungus-growing termite symbiosis and schematic representation of nest structure. (B) Isolation location by bacterial genera; colors mirror the corresponding location in panel A. Ac, *Actinomadura*; Am, *Amycolatopsis*; Lu, *Luteimicrobium*; My, *Mycolicibacterium*; No, *Nocardia*; St, *Streptomyces*. (C) Relative abundances (box plot showing medians, 1st and 3rd quartiles, and all data points) of the six bacterial genera, to which our genomes belong, in previously published 16S rRNA amplicon sequencing community data sets from 10 *M. natalensis* colonies, totaling 10 comb and 86 gut samples (14). These were determined by BLASTn of the 16S rRNA in the termite-associated *Actinobacteria* genomes against a database of the amplicon sequencing community data. Successful hits were set at genus level (95%) and E value ≤ 0.01. Relative abundances were on average significantly higher in combs than guts (*P* = 0.0004; Mann-Whitney U test). Note that DNA extraction protocols tend to bias against Gram-positive bacteria; thus, actual relative abundances are conceivably higher than reported (42).

Termites in the subfamily Macrotermitinae (Termitidae: Blattodea) cultivate basidiomycete fungi in the genus *Termitomyces* (Agaricales: Lyophyllaceae) as their sole food source, using plant material foraged on by the termites (16). The termites, through the symbiosis, manage to fully utilize plant substrates (17) and maintain monoculture fungal farms in densely populated colonies of often millions of termite workers without apparent problems with infectious disease (18). Older workers collect plant biomass (Fig. 1A.1), which is brought back to the nest, where younger workers ingest the substrate along with asexual spores of *Termitomyces* produced in specialized nodules (Fig. 1A.2) and deposit the fecal matter as fresh comb. After this first gut passage, *Termitomyces* grows on the macerated plant substrate within the fungus comb (fungus garden [Fig. 1A.3/4a]). When the plant material is fully utilized, older workers ingest and digest the mature fungus comb (Fig. 1A.4c), after which all organic material is essentially utilized (19). A plethora of bacterial symbionts have been identified from fungus-farming termites, many of which facilitate metabolism of plant and fungal biomass (e.g., *Alistipes*, *Bacteroidetes* [20]) and defense against antagonists (e.g., *Bacillus* [21]). Recent studies have demonstrated the consistent presence of *Actinobacteria* in both termite guts and fungus combs, and the propensities for enzyme and antimicrobial production make them promising candidate plant decomposers and defensive symbionts.

Actinobacterial contributions to defense and plant biomass decomposition in this symbiosis are most likely to occur within combs for three main reasons (15, 22, 23). First, the initial gut passage is rapid (24) and involves minimal plant decomposition (17; but see reference 25 regarding lignin cleavage), so enzyme contributions from *Actinobacteria* during gut passage are unlikely to be important. Second, although gut bacteria would be in a prime position to suppress competitors or antagonists of *Termitomyces* or the termites present in the plant substrate (26), recent findings in Macrotermes bellicosus refuted this “gut sanitation” hypothesis, as potential fungal antagonists appear to pass the gut unharmed and indeed enter fungus combs (27). Lastly, *Actinobacteria* are consistently present and more abundant in fungus combs (14) than guts (Fig. 1C). Thus, *Actinobacteria* contributions are conceivably in fungus combs, while their presence in termite guts is more likely due to ingestion from the plant substrate, soil, or the comb (14, 28, 29).

To elucidate putative functions of *Actinobacteria* in the symbiosis, we comparatively analyze whole-genome sequences of 16 isolates from the fungus-farming termite Macrotermes natalensis and characterize their antimicrobial and carbohydrate-active enzyme (CAZyme) potential. These isolates were obtained from either termite workers (cuticle, gut) or fungus combs (Fig. 1B). To place them phylogenetically, and to provide indications of genome adaptations to symbioses, we compare their genomes with those of the closely related soil-dwelling free-living counterparts and analyze their prophylactic and metabolic potential through whole-genome targeted mining.

## RESULTS

### Phylogenetic placement and genome quality.

**FIG 2:**
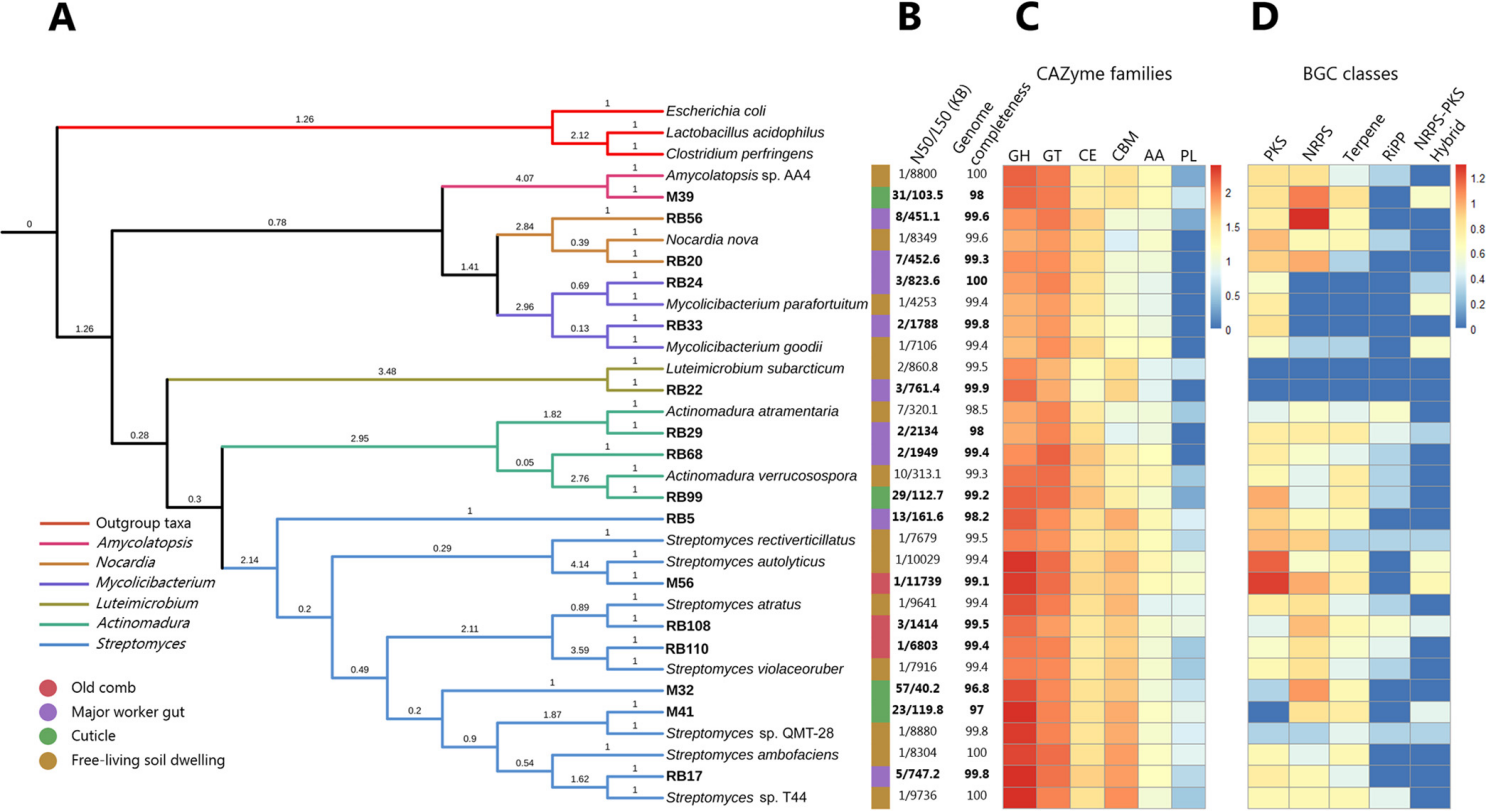
Phylogenetic placement, genome characteristics, CAZymes, and BGCs of the 16 termite-associated *Actinobacteria*. (A) Consensus maximum-likelihood distance tree based on 122 orthologous gene trees with nonparametric bootstrapping, placing the 16 termite-associated bacteria (seen in bold) alongside closely related soil-dwelling relatives. (B) *N*_50_, *L*_50_, and genome completeness (see Table S1 for the full results). *N*_50_ = number of contigs making 50% of the assembly; *L*_50_, length lower limit of contigs making up 50% of the assembly. (C) Log_10_-transformed abundance of CAZymes identified across the genomes. GH, glycoside hydrolase; GT, glycoside transferase; CBM, carbohydrate-binding module; CE, carbohydrate esterase; AA, auxiliary activities; PL, polysaccharide lyase. For full results, see Table S3. (D) Log_10_-transformed abundance of BGCs identified across the genomes. NRPS, nonribosomal peptide synthase; PKS, polyketide synthase; hybrid, NRPS-PKS hybrid; terpenes; and RiPPs, ribosomally synthesized and posttranslationally modified peptides. For the full results, see Table S2.

We first performed multilocus sequence typing (MLST) analyses based on 122 genes to obtain phylogenetic placement of the genomes, and most termite-associated isolates showed short phylogenetic distance to closely related soil species with reference genomes in RefSeq (determined by BLASTn of the 16S rRNA sequence) (Fig. 2). This suggests that the isolates are not specific to being in symbiosis with the termites but more likely originate from the mound or surrounding soil. The quality of the genomes, while varying, was generally very good, with high completeness, high *L*_50_ (length lower limit of contigs making up 50% of the assembly values), long largest contigs, and low *N*_50_ (number of contigs making up 50% of your assembly) values (Fig. 2; see also Table S1 in the supplemental material).

10.1128/mSphere.01233-20.4 TABLE S1 Termite-associated *Actinobacteria* genome metadata and statistics. *N*_50_, number of contigs making 50% of the assembly; *L*_50_, length lower limit of contigs making up 50% of the assembly. Download Table S1, XLSX file, 0.02 MB. Copyright © 2021 Murphy et al. 2021 Murphy et al. https://creativecommons.org/licenses/by/4.0/ This content is distributed under the terms of the Creative Commons Attribution 4.0 International license.

10.1128/mSphere.01233-20.5 TABLE S2 (Sheet 1) Predicted BGCs identified by antiSMASH with a similarity to a reported BGC in the MIBiG database of 50% or higher. The class of BGC, the similar compound identified in the MIBiG database, and whether it has documented antimicrobial activity are detailed. The number relating a sample to a BGC indicates the occurrences of this BGC being found in that sample. (Sheet 2) All BGCs identified by antiSMASH, both novel and predicted (regardless of similarity). Download Table S2, XLSX file, 0.05 MB. Copyright © 2021 Murphy et al. 2021 Murphy et al. https://creativecommons.org/licenses/by/4.0/ This content is distributed under the terms of the Creative Commons Attribution 4.0 International license.

10.1128/mSphere.01233-20.6 TABLE S3 Overview results of HotPep-identified CAZymes. CAZy prediction summary indicating the EC number and corresponding enzyme name of CAZymes identified by HotPep, along with the enzyme substrate, category of enzyme (hydrolase, oxidoreductases, etc.), and whether the enzyme is catabolic or anabolic in nature. The number relating a sample to a specific enzyme is the frequency count of that enzyme in that sample. Download Table S3, XLSX file, 0.04 MB. Copyright © 2021 Murphy et al. 2021 Murphy et al. https://creativecommons.org/licenses/by/4.0/ This content is distributed under the terms of the Creative Commons Attribution 4.0 International license.

### Biosynthetic potential.

**FIG 3:**
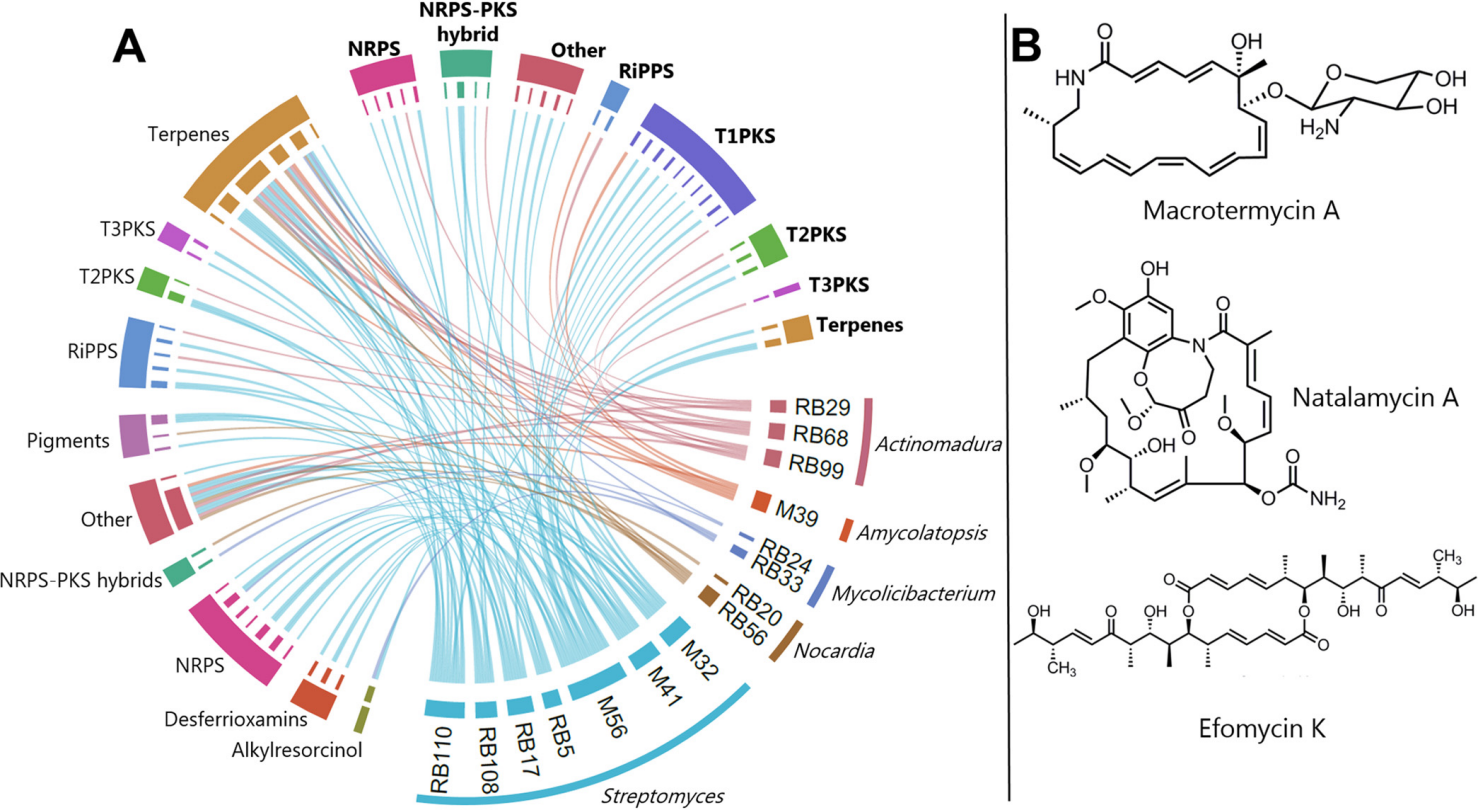
The 16 termite-associated *Actinobacteria* code for diverse BGCs, some of which are shared across isolates. Circular representation of the 112 identified BGCs, based on known biosynthetic gene clusters identified by antiSMASH with 50% or greater similarity to gene clusters in the MIBiG database. Each segment represents a unique BGC or strain with a link between them indicating that the BGC was identified in the strain. Names of BGCs with known antimicrobial activity are highlighted in bold. For the full results, see Table S4. (B) Structures of three recently identified secondary metabolites. The T1PKS-derived natural products natalamycin A and efomycin K were identified in the broad-range antifungal strain M56, although their role in the symbiosis remains unknown (59, 60). The T1PKS-derived macrotermycin A, identified from M39, has selective antifungal activity against *Pseudoxylaria* antagonists in the symbiosis (22).

A total of 435 biosynthetic gene clusters were identified via whole-genome mining by antiSMASH (30, 31) from termite-associated *Actinobacteria*, including 329 with a predicted similar gene cluster in the MIBiG database (32) and 106 putatively novel biosynthetic gene clusters (BGCs) (Table S2). Novel is defined here as no (i.e., 0%) homology to any BGCs in the MIBiG database. Of the 329 BGCs, 116 (65 unique) BGCs were ≥50% similar to a gene cluster in the MIBiG database. A similarity of ≥50% suggests likely similar function to the putative homologue in the MIBiG database. There were 24 unique BGCs encoding compounds with and 42 without known antimicrobial activity (Fig. 3; Table S2), based on the current literature. Unique is defined as a distinct cluster identified by antiSMASH. The total number of BGCs encoding the production of compounds with (25) and without (91) known antimicrobial activity differs considerably from the unique numbers (Table S2). This is likely due to the higher frequency of unique BGCs encoding enzymes that produce compounds without antimicrobial activity being present multiple times across the genomes (Fig. 3) or to the antimicrobial activities of many BGCs having yet to be discovered.

10.1128/mSphere.01233-20.7 TABLE S4 All essential genes identified with ARTS and their frequency of marker occurrence (duplication, proximity to a BGC, horizontal gene transfer, and whether they are a known target). Essential genes are ordered into categories. First, “Most likely resistance factor or target” indicating that in an individual genome for this gene, three or more markers were positive, one being proximity to a BGC. Second, “Likely resistance target or factor,” requiring the same as the previous category, just without proximity to a BGC being one of the markers. Lastly, “Unlikely resistance target or factor,” where fewer than three markers were identified in association with this essential gene in any singular genome. Download Table S4, XLSX file, 0.3 MB. Copyright © 2021 Murphy et al. 2021 Murphy et al. https://creativecommons.org/licenses/by/4.0/ This content is distributed under the terms of the Creative Commons Attribution 4.0 International license.

One BGC that encodes the production of a compound with known activity and 15 BGCs encoding production of compounds without known activity were represented more than once across the isolates; for example, the BGC responsible for the production of ectoine was identified in 12 of the 16 genomes. Only melanin and alkylresorcinol were identified multiple times within a single isolate (twice in M41, RB17, and RB110-1 [henceforth known as RB110] and twice in RB33, respectively). With most BGCs being present in only one isolate, we see the genetic basis for the production of a diverse range of compounds with antimicrobial activities and potentially various modes and ranges of action. Ten of the BGCs encoding the production of natural products with antimicrobial activity have documented antifungal activities, 23 have bacteriostatic or bactericidal effects, two have antiviral effects, and several have antiprotozoal, anticoccidial, and general antiparasitic effects (Table S2). Many modes and ranges of action remained unknown, suggesting ample potential for discoveries of novel bioactivities.

Free-living *Actinobacteria* displayed similar BGC capacities and distributions, with 21 unique BGCs coding for enzymes that produce compounds with known activity and 41 unique BGCs for enzymes without known activity. The total number of BGCs coding for enzymes that produce compounds with and without known activity was comparable to those of termite-associated genomes (260 with versus 86 without). Three BGCs (melanin, alkylresorcinol, and fluostatins M to Q) were identified more than once in a single genome. However, origin of a genome (i.e., termite-associated versus free living) was not significant in predicting BGC class frequency in generalized linear models (glm) when comparing residual deviances (glm, *P* = 0.798) (residual deviance = 306.65 [df = 280] versus residual deviance = 306.59 [df = 279]).

A Dunn test for multiple comparisons, aiming to identify different frequencies of BGC class across termite-associated isolates, showed evidence for terpene enrichment compared to every other BGC class, apart from comparing terpenes to nonribosomal peptide synthases (NRPSs). There was no evidence for enrichment of NRPSs compared to other BGC classes. However, apparent terpene enrichment may be an artifact of contig assembly length, which could cause enrichment of terpene clusters due to their shorter lengths than other major BGC classes (Fig. S2) (33). Four of the five most abundant BGCs that passed our 50% threshold indeed encoded terpenes (Table S2). The BGC encoding the production of geosmin, a sesquiterpene alcohol that contributes to the distinctive smell of soil (34), alone accounts for 10 of the 112 BGCs. This is substantial given that multiple occurrences of BGCs are infrequent (only five were observed four or more times). The tricyclic sesquiterpene albaflavenone was the most frequently encoded compound with known antimicrobial activity. Albaflavenone has shown activity against Bacillus subtilis and is conserved in *Streptomyces* (35, 36).

10.1128/mSphere.01233-20.3 FIG S2 Length (bp) of BGCs with a similarity score of 50% or greater to a known BGC in the MIBiG database (the same BGCs as in Fig. 3). Download FIG S2, TIF file, 1.8 MB. Copyright © 2021 Murphy et al. 2021 Murphy et al. https://creativecommons.org/licenses/by/4.0/ This content is distributed under the terms of the Creative Commons Attribution 4.0 International license.

To explore the potential of unidentified antimicrobials present in the genomes, we utilized Antibiotic Resistance Target Seeker 2 (ARTS2), which identified 5,298 (422 unique) essential genes in the 16 termite-associated *Actinobacteria* (Table S3) through protein family (from the TIGRFAM database) homology to a reference set of core genes from related taxa (37, 38). For a gene to be accepted as a probable resistance factor or target, we determined it would need three of the four associated metadata markers (duplication, proximity to a BGC, known resistance factor/target, and horizontal gene transfer). Following this, 94 (52 unique) genes were categorized as probable resistance factors or targets (Table S3). Among these, 85 (50 unique) were located in close proximity to a BGC identified by antiSMASH. The frequency of metadata markers was not uniform across the genes that passed our threshold (Table S3). Duplication and horizontal gene transfer were the most prevalent markers for potential resistance genes or targets, with a mean relative frequency of 0.344 (±0.0535) and 0.426 (±0.0457), respectively. Comparative analysis showed that the addition of origin (termite-associated versus free-living) did not affect the residual deviance of a generalized linear model, indicating no difference between origins (glm, *P* = 0.061) (residual deviance = 322.37 [df = 459] versus residual deviance = 318.85 [df = 458]). Consistent with this, no apparent differences were observed between the frequency of markers by origin (Fig. S1; Table S3). This suggests that the antimicrobial potential is overall very similar between origins, consistent with the antiSMASH finding of comparable number and activities of BGCs across the genomes.

10.1128/mSphere.01233-20.2 FIG S1 Frequency of essential gene markers produced by ARTS to indicate said gene being a resistance factor or resistant target. Each point is an identified essential gene, with three or more markers, observed in at least one termite-associated bacterial genome. The *y* axis is the frequency of how often that marker is positive in that specific essential gene across all genomes in which the essential gene was identified. Both axes have been jittered to improve visualization. “FGT” denotes that strains are fungus-growing termite-associated, and “free” indicates that strains are closely related soil-dwelling free-living bacteria. Download FIG S1, TIF file, 1.8 MB. Copyright © 2021 Murphy et al. 2021 Murphy et al. https://creativecommons.org/licenses/by/4.0/ This content is distributed under the terms of the Creative Commons Attribution 4.0 International license.

### Metabolic potential of *Actinobacteria*.

**FIG 4:**
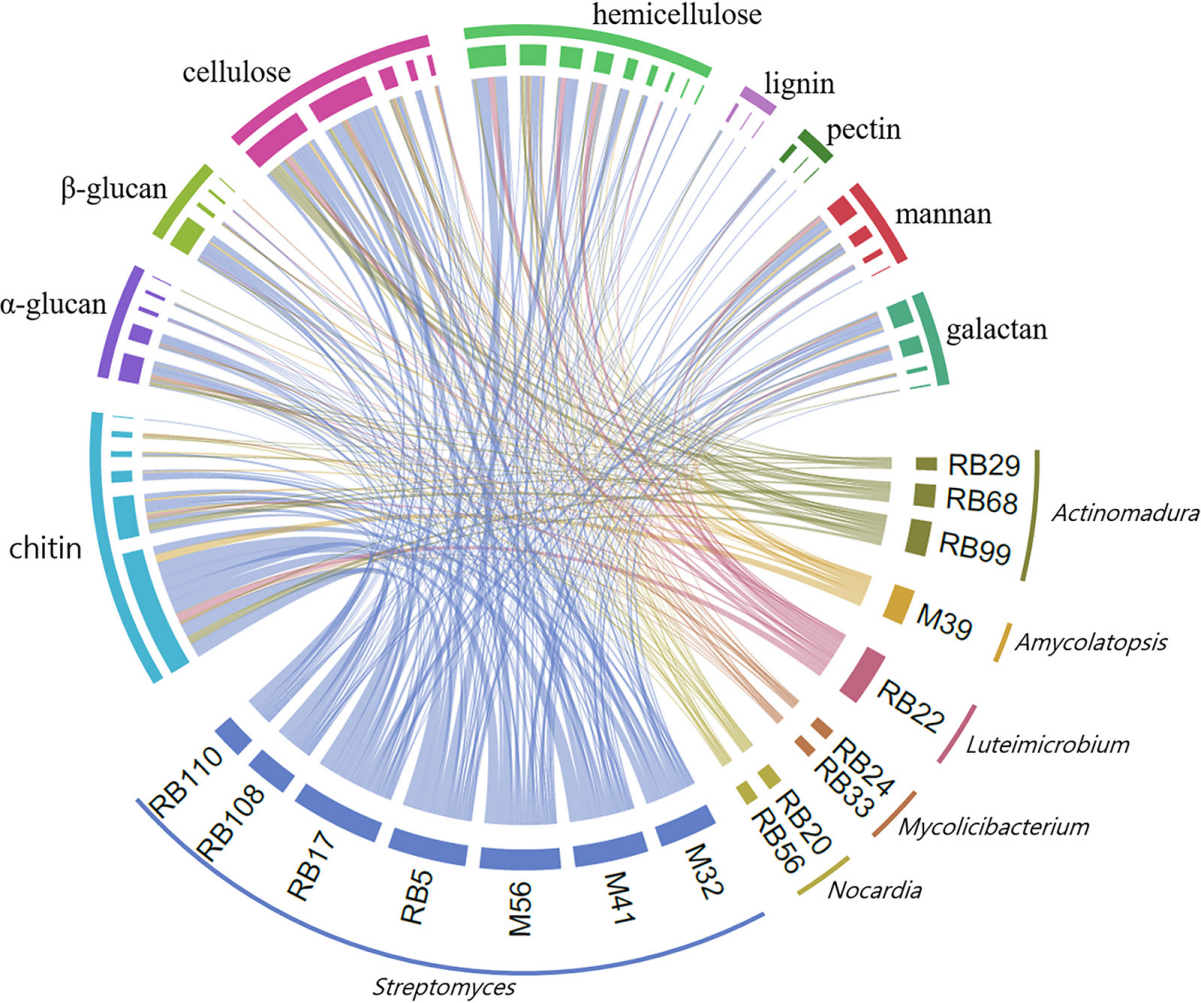
Diverse catabolic CAZymes are distributed across the 16 termite-associated *Actinobacteria*. Circular representation of the 43 unique catabolic plant and fungal cell wall-targeting CAZymes (total 823) sorted by target substrate. Each individual segment represents a unique CAZy gene or strain with a link between them indicating the identification of the CAZy gene in the strain. For the full results, see Table S4.

HotPep identified 5,211 CAZymes across the 16 termite-associated genomes through homology to peptide patterns, of which 1,539 could be identified to EC number level, with 130 unique enzymes, comprised of 93 catabolic and 37 anabolic enzymes (Table S4). Ninety-seven of these were present in more than one genome (Fig. 4). Hydrolases accounted for 92% of all catabolic enzyme types, with 74 unique and 1,108 total enzymes (Fig. 4; Table S3). The predicted substrate targets of these were dominated by chitin, cellulose, and then hemicellulose, vital plant and fungal cell wall components (20) (Fig. 4; Table S3). Seven unique enzymes targeting chitin were observed 240 times, of which chitinase (EC 3.2.1.14) accounted for 65%. Notably, strains were often isolated on chitin medium, so this could bias this analysis. Enzymes cleaving cellulose and hemicellulose were observed on five and 10 unique occasions, with a total of 189 and 157 hits, respectively (Table S4). Cellulase and beta-glucosidase accounted for 40.7% and 41.7% of the cellulose-degrading enzymes, respectively, while acetylxylan esterase and endo-1,4-beta-xylanase accounted for 21% and 29% of hemicellulose-degrading enzymes, respectively. Termite-associated strains were not significantly different from free-living strains in their predicted degradative potential (glm, *P* = 0.253) (residual deviance = 1,020.3 [df = 1,409] versus 1,020.8 [df = 1,410]).

## DISCUSSION

*Actinobacteria* are consistently present as low-abundance members of *M. natalensis* fungus combs, and to a lesser extent in gut microbiomes (Fig. 1C) (13). However, despite their low abundance, precedents from other work support that low-abundant taxa can be of significant importance (39–41). Furthermore, our current perception of their abundances in the termite symbiosis is likely to be underestimated, DNA extraction techniques tend to be biased against Gram-positive bacteria (42). Our analyses identified BGCs and CAZymes that may be of importance for the symbiosis, potentially with *Actinobacteria* as mutualistic symbionts, and our findings led us to three main conclusions. First, the *Actinobacteria* genomes contain numerous BGCs coding for natural products with potential antimicrobial activity (5, 43), of which a large proportion have yet to be elucidated chemically. This suggests ample potential for discovery of novel antimicrobials. Second, the genomes contain genes for enzymes targeting all plant and fungal substrates identified in fungus-growing termite gut community-level analyses (20). Lastly, although *Actinobacteria* represent a very small set of the complex microbiomes present within termite guts and fungus combs, their large enzymatic potential is consistent with their well-established roles in natural ecosystems as efficient biomass degraders (16, 20, 44–47) and prolific producers of antimicrobial compounds (5, 43). In line with this, the closely related free-living *Actinobacteria* show similar metabolic and enzymatic potential, thus making horizontal acquisition highly likely. In light of these findings, we discuss the potential for *Actinobacteria* serving roles in associations with farming termite hosts.

The persistent similarities in genomes irrespective of their origin being from termite colonies or free living suggest that the termite-obtained *Actinobacteria* do not display unique genomic signatures that point to specialized biomass decomposition or antimicrobial production. This could suggest that they may randomly colonize fungus combs or termites from the substrate harvested by the termites, from ingested soil (14, 28, 29, 48), or from contact with the surrounding soil. Although the net positive (mutualistic) benefit to the termites is currently not known, their presence implies potential effects in the comb environment, and considering that they are not removed by the termites, it makes it unlikely that they negatively impact the termite-*Termitomyces* association. The termites could then have coopted members of the phylum as symbionts because of their degradative or biosynthetic potential, without specific selection leading to apparent fungal genomic changes. This would mirror many other ectosymbiotic associations that have arisen because of symbiont (or host) metabolic capacities, but without genome erosion that is mostly apparent in strictly vertically transmitted endosymbionts (49–52). Furthermore, it appears to be only a subset of *Actinobacteria* in the surrounding soil that are capable of colonizing the symbiosis. *Actinobacteria* is one of the most abundant soil phyla (53–55), yet its members remain low in abundance within the symbiosis. To distinguish whether these are consistent colonizers of fungus-growing termites, we need to examine their presence in surrounding soils and within the symbiosis across nests.

If *Actinobacteria* symbiotically contribute to plant biomass decomposition by lignocellulolytic enzymes, this is most likely to occur within the comb environment (15, 22, 23) for two reasons. First, the predigestive gut passage of the substrate for fungiculture is comparably fast (Fig. 1A.1), and the relatively low abundance of the bacteria makes it unlikely that they play a major role at this stage of decomposition, despite their extensive cellulose- and hemicellulose-degrading capabilities. This is consistent with previous findings that cellulose, hemicellulose, and lignin-rich material enter the fungus comb in *M. natalensis* (17), within which *Termitomyces* dominates as the plant biomass degrader (16, 17, 56). Lignin may in part be cleaved during gut passage in the fungus-farming termite species Odontotermes formosanus (25), but although lignin-degrading enzymes such as laccase (EC 1.10.3.2) are extensively present in the *Actinobacteria* phylum (57), these occur infrequently in our isolates. Detailed gene expression analyses of combs to targeting both *Termitomyces* and bacterial communities remain to be performed but could provide insights into their relative contributions.

Maintaining monocultural fungus farming for years without other fungi present until colonies are compromised by external factors or succumb after the royal pair dies (58) requires highly effective defense functions. *Actinobacteria* have been proposed to play roles in facilitating these disease-free conditions through antimicrobial production (13, 22, 23, 59, 60). Our genome analyses of the termite-associated strains corroborate this promising defensive potential and detail a diverse set of NRPS-, PKS-, and terpene-related BGCs that are putative or known antimicrobials. The dominant compounds encoded by BGCs in both termite-associated and free-living genomes are common to *Actinobacteria* and not yet known to be antimicrobial (61–63). The potential broad spectrum of the diverse set of BGCs, supported by knowledge of compounds with known ranges and modes of action, may suggest an unselective antimicrobial landscape that could contribute to suppressing a series of targets. The most plausible site of *Actinobacteria* contributions is again within the fungus comb environment within which potential competitor or antagonist fungi might thrive (27).

One of our most striking findings was the high abundances of mycolytic enzymes targeting key fungal cell wall subunits encoded in the genomes. While termite gut microbes harbor large fungal biomass degradation potential (20, 25), the presence of these enzymes in predominantly comb-residing bacteria may appear counterintuitive. This could play a nutritional role; however, digestion of fungus material is not expected within fungus combs before ingestion by the termites. Alternatively, chitinase production could act as a layer of antifungal defense within combs if applied directly to sites of fungal infection by the termites, potentially targeting non-*Termitomyces* fungi. However, a perhaps more plausible explanation is that chitinases are ubiquitous in the comb and indiscriminately degrade fungal call wall within the comb, resulting in a potential cost of association for the termites and *Termitomyces*. This, however, would then potentially be mitigated by their low abundance and the persistent seeding and high abundance of *Termitomyces* or be outweighed by the putative antimicrobial benefits that *Actinobacteria* may provide.

The variety of antifungals identified within this study combined with the *in vitro* insights from previous bioassay reports (15) and tests of several specific isolates (M39, M56, RB108, and RB29 [13, 22, 23, 59, 60]) further supports unselective nest defense. Although a diverse range of fungal genera enter fungus combs after plant substrate passes the termite gut (27), their extremely low abundance within combs implies very effective growth suppression. Thus, further experimentation would be required to determine if seeding rate and general abundance of *Termitomyces* are what allows for monoculture with minimal presence of other fungi. Counter to this is identification of macrotermycins in *Amycolatopsis* M39, a putatively selective inhibitor of the stowaway fungus *Pseudoxylaria* (22), suggesting that a combination of targeted and untargeted approaches may be employed.

Our analyses of 16 termite-associated actinobacterial genomes also aimed to elucidate the genetic potential for the production of novel natural products and revealed a surprising number of BGCs with low/no homologies to previously reported BGCs. Our addition of ARTS2 resulted in the identification of an additional 50 essential genes that pass the three-marker threshold, with one of those markers being close proximity to an identified BGC. This remarkable finding speaks for the high potential to identify not only new bacterial diversity (64–66) but also novel natural products. Further work on both gene expression and compound production within termite colonies as well as biochemical studies to characterize the underlying gene clusters and their products is needed to further elaborate the roles of *Actinobacteria* within the fungus-farming termite system.

## MATERIALS AND METHODS

### Genomes, assembly, and annotation.

Ten of the *Actinobacteria* genomes (RB29, RB68, RB99, M39, RB20, RB56, M41, M56, RB5, and RB17) were previously isolated from workers (cuticle, gut) or fungus combs (Fig. 1B) of the fungus-farming termite species *Macrotermes natalensis*, and genomes were downloaded from NCBI (see Table S1 in the supplemental material). We complement this set of genomes from the literature with five additional isolates that we sequence as part of this work; these have been deposited at NCBI (Table S1). The strains occupy the Actinomycetales order, spanning three suborders, six families, and six genera (Table S1).

Three of the six new strains (RB22, RB24, RB33, and RB108) were isolated from worker guts (RB22, RB24, and RB33) and two from fungus comb (RB108 and RB110-1 [referred to here as RB110]) of *M. natalensis*, and genomic DNA was extracted from cultures grown in ISP2 broth on a rotary shaker at 150 rpm at 30°C. Cells were harvested, and genomic DNA was extracted using the GenJet genomic DNA purification kit (Thermo Scientific, catalog no. K0721) following the manufacturer’s instructions, except with the following changes: lysozyme and proteinase K treatments were each extended to 40 min. For the Illumina library prep, genomic DNA was sheared using a Covaris S220 sonication device (Covaris Inc., MA, USA). Sequencing was performed using NEBNext Ultra II (New England Biolabs, Frankfurt, Germany) paired-end libraries on a MiSeq sequencer (RB22, RB24, RB33, and RB108) or NovaSeq sequencer (RB110). Quality trimming and adapter clipping were performed using Trimmomatic v.0.36 (67). Additional rounds of adapter clipping and filtering of low-complexity reads were performed using BBDuk of the BBTools package (68) v.36.84 (https://sourceforge.net/projects/bbmap/) and cutadapt (69) v.1.13 (https://github.com/marcelm/cutadapt). Overlapping read pairs were merged using FLASH (70) v.1.2.11 (https://ccb.jhu.edu/software/FLASH/). High-molecular-weight (HMW) DNA for PacBio-based whole-genome sequencing of RB110 was extracted using the NucleoBond HMW DNA kit (Macherey-Nagel).

Genomic DNA of *Streptomyces* sp. strain M32, isolated from an *M. natalensis* worker cuticle, was obtained from a 50-ml overnight culture at 30°C in ISP2. Extraction of DNA was performed using the GenElute bacterial genomic DNA kit (NA2100; Sigma-Aldrich), and the genome was sequenced at the Harvard Medical School Biopolymers Facility using the HiSeq2000 flow cell (Illumina CASAVA 1.8.2). Sequencing was performed using Illumina TruSeq 50-bp paired-end libraries on a HiSeq 2000 sequencer.

Because the new and downloaded genomes were compiled from different studies, assembly and annotation techniques and versions vary (see Table S1 for the full details). All Illumina MiSeq genomes (RB5 to -108) were assembled using SPAdes (71) v3.10.1 (https://github.com/ablab/spades), except RB29, for which v3.6.29 was used. M32 and M39 (22) were assembled with the A5 pipeline (72) v.20120518 (https://sourceforge.net/p/ngopt/wiki/A5PipelineREADME/). All assemblies were annotated with Prokka (73), with M56 and RB5 to -108 (except RB20, RB22, and RB56) utilizing v1.11 and the rest utilizing v1.12 beta. RB110 was hybrid assembled following a custom procedure. PacBio long reads were *de novo* assembled via Canu (74) v2.1.1 (https://github.com/marbl/canu) with default settings. This assembly was polished with the NextPolish pipeline v1.3.1 (75) using the quality-controlled (to a Phred score of 30) and merged Illumina NovaSeq and PacBio Sequel reads; see Text S1 in the supplemental material for details. The final assembly was annotated using Prokka (73) v1.14.5. RB22, RB24, RB33, and RB110 assemblies and annotated GenBank files were deposited to Zenodo (https://doi.org/10.5281/zenodo.4302144).

10.1128/mSphere.01233-20.1 TEXT S1 Scripts used in biosynthetic gene cluster analysis and CAZyme and phylogenetic analysis, including statistical analysis. Consult the README file for specifics on how to run each analysis pipeline. All large-scale computation was performed on an HPC Conda environment manager with Slurm queue systems. Download Text S1, TXT file, 0.04 MB. Copyright © 2021 Murphy et al. 2021 Murphy et al. https://creativecommons.org/licenses/by/4.0/ This content is distributed under the terms of the Creative Commons Attribution 4.0 International license.

To determine if termite-associated *Actinobacteria* differ genetically from free-living counterparts, we downloaded 14 high-quality genomes of the closely related free-living strains (based on BLASTn of the 16S rRNA sequence) from the NCBI RefSeq database (Table S1). All genomes of free-living isolates were annotated with Prokka (73) v1.14.5.

### Phylogenetic placement of strains.

Universal single-copy orthologous genes for all genomes were identified with BUSCO v4 (76) with default settings and autolineage flag engaged. Counts for all 130 genes in the BUSCO bacterial data set were determined, and multi-Fasta files were generated for ortholog genes present in three or more genomes and then aligned with Clustal Omega v1.2.4 (77). Phylogenies from the multilocus sequence typing (MLST) using 122 orthologs were generated using RAxML-NG v0.9.9 (78), employing the –all mode, GTR+G model, and a seed of 2. Branch support based on bootstrapping and transfer distance were obtained, before combining the gene trees into an unrooted species tree using ASTRAL-Pro v1.12 (79).

### Biosynthetic potential and resistance mechanisms.

Identification of the biosynthetic potential of *Actinobacteria* was carried out using antiSMASH v.5 (30, 31). The known clusters, subclusters, general, full HMMER, and pfam2go flags were utilized to generate a maximum output for a single run. Custom scripts were created to extract the known clusters information, that being biosynthetic gene clusters (BGCs) in which a similar compound was identified in the MIBiG database (https://mibig.secondarymetabolites.org/) along with the class of BGC (e.g., NRPSs, PKSs, terpenes, RiPPs [ribosomally synthesized and posttranslationally modified peptides], etc.). Only BGCs with ≥50% similarity score to the MIBiG database were retained to ensure likely similar functionality between homologous BGCs. A description of each compound was obtained via manual searching of the literature, including the mode of action and range of activity for compounds known to have antimicrobial properties. Comparative analysis of biosynthetic gene cluster class was undertaken by modeling the effects of isolate origin, either termite-associated or most closely related soil-dwelling free-living, on the occurrence of BGC class through Poisson distributed generalized linear models (glm) (from base R v4.0 [80]) with and without origin.

To explore the potential of unknown antimicrobial activity present in the genomes, we utilized Antibiotic Resistance Target Seeker (ARTS) version 2 (38). Prokka-annotated GenBank files with sequences for all genomes were run through the tool, which automates target-directed genome mining and builds on the concepts set out by Wright et al. (82) to exploit self-protection mechanisms inherent with the production of antimicrobially active compounds (37). This allowed searches for known resistance factors/targets and identification of potentially novel factor/targets through screening for duplications of essential genes, proximity to detected biosynthetic gene clusters (through antiSMASH), and whether a gene is likely to be horizontally transferred. These markers allow for inference of the essential gene being associated with self-protection and, thus, indicate antimicrobial activity of a gene-encoded natural product (37, 38). Essential genes are possible drug targets defined on a basis of ubiquity within a semibroad phylogenetic scope. Using Poisson-distributed generalized linear models, the results of ARTS2 were used to identify if origin (termite-associated versus soil-dwelling free-living) affected resistance factor/target marker counts associated with essential genes in a manner similar to the antiSMASH result modeling.

### Metabolic potential.

Identification of carbohydrate-active enzymes (CAZymes) was carried out via Homology to Peptide Pattern utilized by the HotPep tool (https://sourceforge.net/projects/hotpep/) using CAZyme PPRY patterns v.1. Following this, all identified CAZymes with an EC number to a specific enzyme were annotated with an enzyme name using the ExPASy enzyme.dat database (ftp.expasy.org/databases/enzyme) accessed on 17 February 2020. The overarching categories (lyases, transferases, hydrolases, etc.) of each enzyme were determined by taking the highest level of the corresponding EC number and searching the Brenda online database (https://brenda-enzymes.org/). The substrate of each enzyme was determined via manual searching of the Brenda and KEGG databases (http://www.genome.jp/kegg/). We comparatively analyzed summaries of counts of enzymes by target substrate by modeling the effect of origin (termite-associated versus free-living) using nested negative binomial distributed generalized linear models, and with substrate as a constant predictor variable in both models (glm.nb from MASS v7.3-51.6 [81]).

### Data availability.

All genomes are publicly available, either from previous publications, submission to the NCBI database under accession number JAEKDS000000000.1 for RB110, or Zenodo at https://doi.org/10.5281/zenodo.4302144 (RB22, RB24, RB33, M32, and RB108). All other accession numbers are provided in Table S1.

10.1128/mSphere.01233-20.8 TABLE S5 Overview of all BGCs previously identified from *Actinobacteria* isolated from the fungus-growing termite symbiosis. Download Table S5, XLSX file, 0.02 MB. Copyright © 2021 Murphy et al. 2021 Murphy et al. https://creativecommons.org/licenses/by/4.0/ This content is distributed under the terms of the Creative Commons Attribution 4.0 International license.
