# Supplementary figures and images for: Comparative Genomics Reveals Prophylactic and Catabolic Capabilities of Actinobacteria within the Fungus-Farming Termite Symbiosis
Source: mSphere. 2021 Mar 3;6(2):e01233-20. doi: 10.1128/mSphere.01233-20 (PMC8546716; doi:10.1128/mSphere.01233-20)

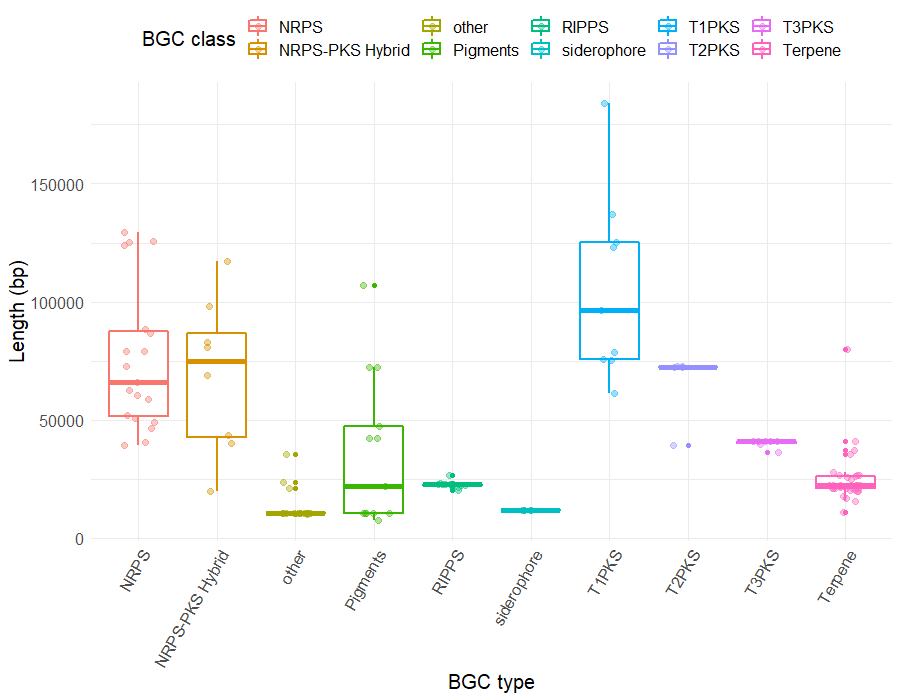

Supplement: FIG S2 [file msphere.01233-20-sf002.tif]

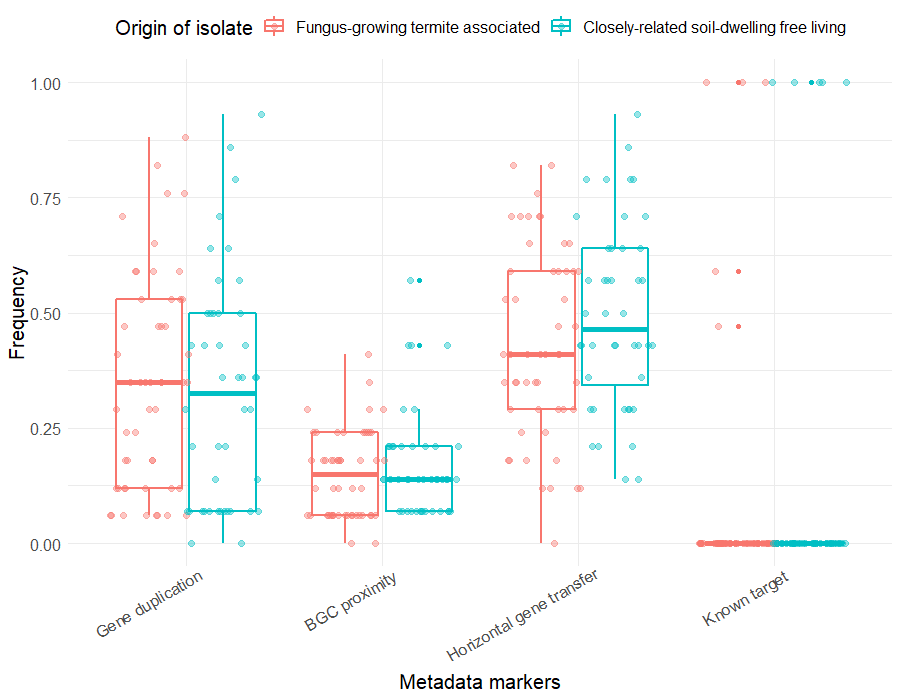

Supplement: FIG S1 [file msphere.01233-20-sf001.tif]
